# CD271 is a functional and targetable marker of tumor-initiating cells in head and neck squamous cell carcinoma

**DOI:** 10.18632/oncotarget.2269

**Published:** 2014-07-26

**Authors:** Oihana Murillo-Sauca, Man Ki Chung, June Ho Shin, Christina Karamboulas, Shirley Kwok, Young Ho Jung, Richard Oakley, James R. Tysome, Lovisa O. Farnebo, Michael J. Kaplan, Davud Sirjani, Vasu Divi, F. Christopher Holsinger, Chafeek Tomeh, Anthony Nichols, Quynh T. Le, A. A. Dimitrios Colevas, Christina S. Kong, Ravindra Uppaluri, James S. Lewis, Laurie E. Ailles, John B. Sunwoo

**Affiliations:** ^1^ Division of Head and Neck Surgery, Department of Otolaryngology, Stanford University School of Medicine, Stanford, CA; ^2^ Department of Radiation Oncology, Stanford University School of Medicine, Stanford, CA; ^3^ Department of Medicine, Stanford University School of Medicine, Stanford, CA; ^4^ Department of Pathology, Stanford University School of Medicine, Stanford, CA; ^5^ Stanford Cancer Institute, Stanford University School of Medicine, Stanford, CA; ^6^ Stanford Institute for Stem Cell Biology and Regenerative Medicine, Stanford University School of Medicine, Stanford, CA; ^7^ Ontario Cancer Institute, University Health Network, Toronto, Canada; ^8^ Department of Otolaryngology – Head and Neck Surgery, Victoria Hospital, Schulich School of Medicine and Dentistry, London, Ontario, Canada; ^9^ Department of Otolaryngology – Head and Neck Surgery, Washington University School of Medicine, St. Louis, MO; ^10^ Department of Pathology and Immunology, Washington University School of Medicine, St. Louis, MO

**Keywords:** HNSCC, cancer stem cells, NGFR, p75NTR, cancer-initiating cell, CD44

## Abstract

Tumor-initiating cells (TICs) in squamous cell carcinoma of the head and neck (SCCHN) are best characterized by their surface expression of CD44. Although there is great interest in identifying strategies to target this population, no marker of these cells has been found to be functionally active. Here, we examined the expression of the purported marker of normal human oral epithelial stem cells, CD271. We show that CD271 expression is restricted to a subset of the CD44^+^ cells. Using xenograft assays, we show that the CD44^+^CD271^+^ subpopulation contains the most tumorigenic cells. Loss of CD271 function results in a block in the G2-M phase of the cell cycle and a profound negative impact on the capacity of these cells to initiate tumor formation *in vivo*. Incubation with recombinant NGF results in enhanced phosphorylation of Erk, providing additional evidence that CD271 is functionally active. Finally, incubation of SCCHN cells with antibody to CD271 results in decreased Erk phosphorylation and decreased tumor formation *in vivo*. Thus, our data are the first to demonstrate that CD271 more specifically identifies the TIC subpopulation within the CD44^+^ compartment in SCCHN and that this receptor is a functionally active and targetable molecule.

## INTRODUCTION

Squamous cell carcinoma of the head and neck (SCCHN) is one of the most common malignant neoplasms of the upper aerodigestive tract. While there have been some modest improvements in the treatment of advanced cases, recurrence rates are still as high as 30-50% for tobacco-related SCCHN, and the therapy alone – which includes a combination of surgery, chemotherapy, and radiation – often results in significant morbidity and long-term functional toxicity, negatively affecting critical functions such as speech and swallowing.

Tumor-initiating cells (TICs) in solid tumors represent a subpopulation of cells that have the ability to propagate tumor formation *in vivo* and have been associated with recurrence of disease and poor clinical outcomes due to their metastatic capacity and resistance to conventional chemotherapy and radiation. In SCCHN, the hyaluronic acid receptor CD44 has reproducibly been shown to be a marker that can distinguish these cells from non-TICs [1]. Specifically, the CD44^+^ population has been shown to contain the TIC subpopulation, since purified CD44^+^ cells from heterogenous primary tumors are able to give rise to tumors much more readily in xenograft model systems compared to CD44^−^ cells, and these xenograft tumors subsequently reproduce the original tumor heterogeneity observed in the primary tumor. Importantly, the CD44^+^ population has also been discovered to have a greater capacity to handle oxidative stress and, as such, is more radioresistant [2]. This population has also been shown to have a significantly greater ability to metastasize to regional lymph nodes in animal models [3], and patients whose tumors have greater percentages of CD44^+^ cells have a significantly poorer clinical outcome [4]. Thus, there has been a strong growing interest in identifying strategies to target these cells. However, the discovery of targetable functional molecules identifying the TICs in SCCHN has remained elusive.

In normal human oral epithelium, a subpopulation of cells with stem cell – like properties has been shown to express a cell surface molecule, designated as the CD271 antigen [5, 6]. This molecule, also known as the low affinity nerve growth factor (NGF) receptor or p75^NTR^, is a neurotrophin receptor and a member of the tumor necrosis factor receptor superfamily. In the nervous system, it has critical functions in cell survival [7], differentiation [8], and migration [9] of neuronal cells. Recently, this molecule has been identified as a marker of TICs in human melanoma [10, 11], esophageal carcinoma [12, 13], and hypopharyngeal carcinoma [14]. In addition to being expressed in discrete cells within the basal layer of normal oral epithelium, CD271 is also expressed in oral dysplasia and oral squamous cell carcinoma [15]. Importantly, the increased expression of CD271 has been associated with a poorer clinical outcome in esophageal cancer [16, 17], hypopharyngeal cancer [14], and oral squamous cell carcinoma [15, 18].

In this study, we show that cells expressing CD271 in human and mouse SCCHN comprise a distinct subset of the CD44^+^ cells and that these CD44^+^CD271^+^ cells possess the greatest tumor-initiating capacity in this malignancy. Further, our data demonstrate that this receptor is functional in SCCHN and that inhibition of CD271 has profound negative effects on SCCHN tumor-initiating capacity, providing evidence for the first functional and targetable molecule specific to TICs in this malignancy.

## RESULTS

### CD271 is expressed in the majority of head and neck SCC

We assessed the prevalence of CD271 expression in head and neck SCC by immunohistochemical staining of a tissue microarray (TMA) containing 283 specimens from primary tumors (Table 1). Overall, 71% of the tumors showed strong positive CD271 staining (representative staining shown in Supplemental Figure 1). No correlation was observed with a particular anatomic site or with clinical parameters, such as TNM staging and outcome. However, these specimens represent a heterogeneous collection of mucosal tumors, including those from the oral cavity, oropharynx, hypopharynx, and larynx. There were a higher percentage of CD271^+^ tumors among the oropharyngeal SCC group of tumors, the majority of which were human papilloma virus positive, but there was no statistically significant difference in CD271 expression by HPV DNA or p16 status (data not shown).

**Table 1:** Expression of CD271 in human primary SCCHN samples measured by immunohistochemistry

	Oropharyngeal (%)	Non-Oropharyngeal (%)	Total (%)
CD271^+^	154 (76)	47 (58)	201(71)
CD271^−^	48 (24)	34 (42)	82 (29)

### CD271 is expressed on a discrete subset of SCCHN cells that have the capacity to initiate tumors

**Figure 1:**
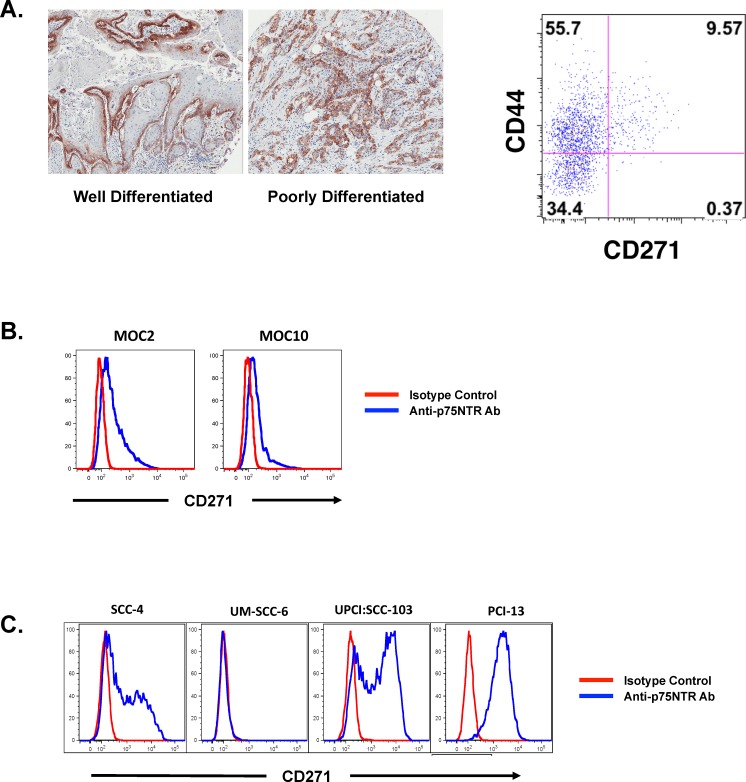
CD271 is heterogeneously expressed in SCCHN and marks the more tumorigenic population of cells (A) Human primary SCCHN samples were stained with a monoclonal antibody against CD271 and assessed by immunohistochemistry and flow cytometry. The FACS plot is gated on the DAPI^−^lineage^−^ population and co-stained for CD44 and CD271. (B) Two murine cell lines derived from oral tumors arising from DMBA-treated oral cavity mucosa were stained with a monoclonal antibody specific for murine CD271 and assessed by FACS. (C) Human cell lines derived from head and neck SCC were assessed for CD271 expression by FACS after staining with a monoclonal antibody to CD271.

The expression pattern of CD271 in SCCHN was interesting in that there was discrete expression of this receptor on a distinct subpopulation of cells in oral SCC. Among well-differentiated oral SCC tumors, the expression of CD271 was in the “basal” aspect of the malignant epithelium, which maintained polarity (Figure 1A). This basal expression is consistent with what has been described in normal human oral epithelium among cells possessing stem cell – like properties [5]. In tumors that were more poorly differentiated, there was more disorganized expression of CD271, but the subpopulation that was positive for the receptor was still distinct and separate from the CD271^−^ cells. This discrete expression pattern was also observed in dissociated primary tumor cells that were stained for CD44 and CD271 and analyzed by flow cytometry. Importantly, the CD271^+^ cells comprised a subpopulation among the CD44^+^ cells in these primary tumors, but there was no appreciable CD44^−^ CD271^+^ subpopulation (Figure 1A). A distinct CD271^+^ subpopulation of cells was also observed in cell lines derived from both mouse and human oral squamous cell carcinoma (Figures 1B and 1C).

Because CD271 has been associated with normal and cancer stem cell phenotypes, we assessed whether CD271^+^ cells had a greater capacity for tumor initiation *in vivo*. Specifically, we were interested in determining whether the CD271^+^ cells possessed the primary tumor initiating capacity among the CD44^+^ population and if the CD44^+^CD271^+^ cells represented the more specific TIC population in SCCHN. To test this, we sorted the CD44^+^CD271^+^ cells, CD44^+^CD271^−^ cells, and CD44^−^ CD271^−^ cells from primary human oral SCC specimens (Supplemental Figure 2) and implanted them into the flanks of immunodeficient Rag^−/−^γc^−/−^ mice in a limiting dilution fashion. Overall, the CD44^+^ cells had greater capacity to form xenograft tumors compared to the CD44^−^ cells, but when low numbers of cells were injected, it was clear that the CD44^+^CD271^+^ cells had the greatest capacity to form tumors among these three populations (Table 2); The tumors that formed in mice implanted with the CD44^+^CD271^+^ cells recapitulated the heterogeneity seen in the parental tumor (Supplemental Figure 3). Similarly, when mouse oral SCC cell lines (which were uniformly CD44^+^, data not shown) were sorted into CD271^+^ and CD271^−^ populations, the CD271^+^ cells had significantly greater capacity to form tumors *in vivo* compared to the CD271^−^ cells(Table 2). Thus, in SCCHN, the TIC population is marked by the expression of both CD44 and CD271.

**Table 2:** Xenograft tumor formation with sorted population of human and mouse SCCHN cells

Human Primary Head and Neck SCC Tumor #1				
Cells Injected	50,000	5,000	500	
CD44^−^CD271^−^		0 of 2	0 of 6	
CD44^+^CD271^−^	2 of 2	5 of 6	0 of 6	
CD44^+^CD271^+^			3 of 5	
Human Primary Head and Neck SCC Tumor #2				
Cells Injected	100,000	10,000	1,000	100
CD44^−^CD271^−^	2 of 2	5 of 6	4 of 6	
CD44^+^CD271^−^	2 of 2	8 of 8	7 of 8	4 of 8
CD44^+^CD271^+^	2 of 2	8 of 8	8 of 8	8 of 8
Mouse Oral SCC Cell Line: MOC2				
Cells Injected	10,000			
CD271^−^	0 of 5			
CD271^+^	3 of 5			
Mouse Oral SCC Cell Line: MOC10				
Cells Injected	10,000			
CD271^−^	0 of 5			
CD271^+^	3 of 5			

### Knockdown of CD271 in SCCHN inhibits cell proliferation and tumor formation

**Figure 2:**
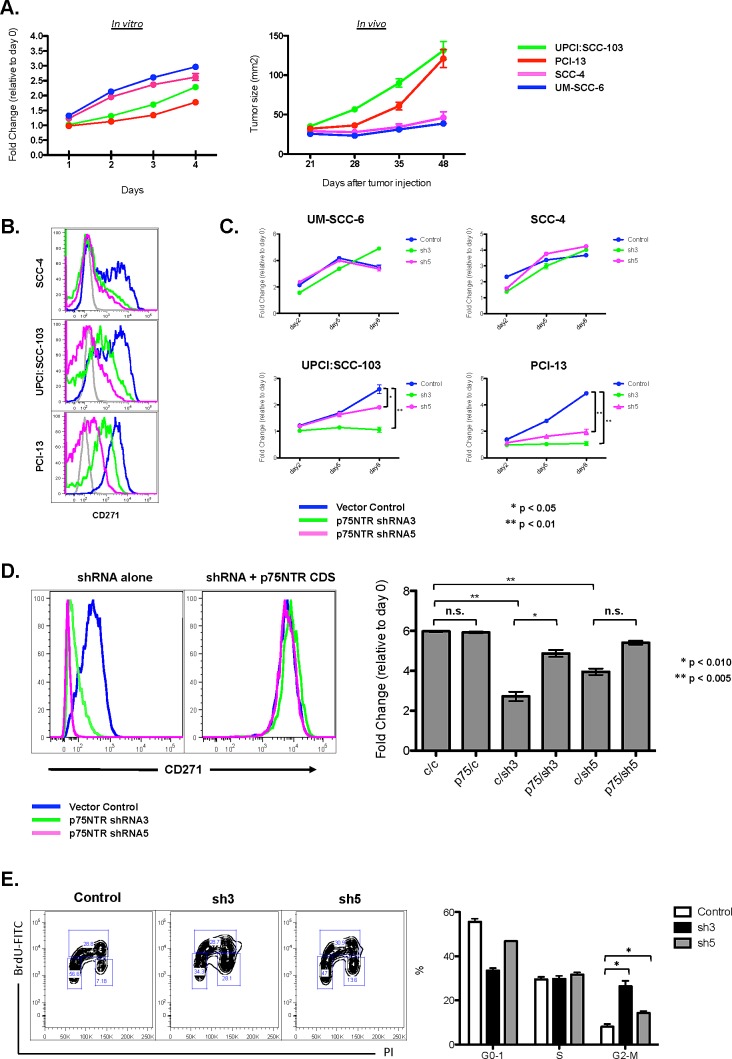
CD271 loss-of-function results in decreased cell cycle progression in SCCHN (A) Four cell lines derived from human SCCHN were assessed for in vitro growth kinetics by MTT assay and for in vivo tumor formation and growth by implantation into the flanks of Rag^−/−^γc^−/−^ mice. (B) Knockdown of CD271 in human SCCHN cell lines by shRNA lentiviral constructs was assessed by FACS. (C) Effects of CD271 knockdown by shRNA expression on cell proliferation was assessed by MTT assay. In the cell lines with high expression of CD271 (UPCI:SCC-103 and PCI-13), there was a decrease in cell viability at the end of the MTT assay. Two different shRNAs were assessed: “sh3” = CD271 shRNA3. “sh5” = CD271 shRNA5. (D) The effects of the CD271 knockdown was reversed by co-expression of the CD271 coding sequence that lacked the 3′-UTR, which was targeted by the shRNA. In the FACS plots, the rescued expression of CD271 is demonstrated in the plot on the right. The bar graph represents the fold change in cell viability at the end of an MTT assay (relative to day 0). “c” = empty vector control or scramble RNA control. “p75”= CD271 coding sequence lacking the 3′-UTR. “sh3” = CD271 shRNA3. “sh5” = CD271 shRNA5 *p<0.01, **p<0.005. (E) PCI-13 cells were transduced with lentivirus expressing CD271 shRNA3, CD271 shRNA5, or a scrambled control RNA. Percentages of cells in the various phases of the cell cycle were assessed by FACS after staining with BrdU and PI; these percentages are quantified in the bar graph. *p<0.01.

As with primary head and neck SCC, the expression of CD271 in human SCCHN cell lines was heterogeneous (Figure 1C). Among the four SCCHN cell lines that we examined, there were varied percentages of CD271^+^ cells. Although these cell lines had similar growth characteristics *in vitro*, implantation of the two lines with the highest percentages of CD271^+^ cells in mice resulted in the most robust *in vivo* tumor growth (Figure 2A). Because CD271 has been shown in other systems to modulate cell proliferation and survival [19-22], we investigated whether or not CD271 loss-of-function would have an effect on cell proliferation and tumor initiation. To do this, we expressed validated shRNA molecules targeting CD271 in SCCHN cells by lentiviral transduction. The knockdown of CD271 significantly affected cell growth *in vitro* (Figure 2C). In an MTT assay, the CD271^hi^ cell lines (UPCI:SCC-103 and PCI-13), that were transduced with the CD271 shRNA, grew significantly more slowly compared to controls. This growth was rescued by co-transducing the coding sequence for CD271, which lacked the 3′-UTR targeted by the shRNA (Figure 2D); thus, the reduced growth conferred by the shRNA was specific for CD271. Mechanistically, this appears to be due to a partial cell cycle block in the G2-M transition and not apoptosis (Figure 2E and Supplemental Figure 4). Thus, for cells expressing CD271, the receptor appears to be required for cell cycle progression.

**Figure 3:**
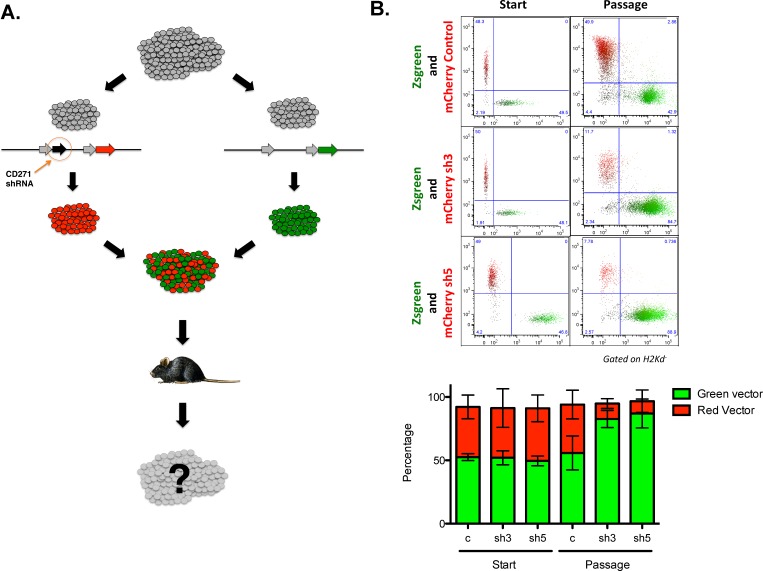
CD271 loss-of-function results in decreased tumor initiation *in vivo* (A) Schematic of *in vivo* competition assay. PCI-13 cells were transduced with either a lentivirus co-expressing shRNA targeting CD271 and mCherry or a lentivirus expressing Zsgreen. Cells were mixed 1:1 and injected into the flanks of Rag^−/−^γc^−/−^ mice. Cell composition in tumors arising in these mice was assessed by FACS analysis of cells from dissociated tumors. (B) FACS analysis of input cells at time of implantation and cells from dissociated tumors at the termination of the experiment. The bar graphs depict the percentages of mCherry and Zsgreen expressing tumor cells from each cohort of recipient mice.

To assess if CD271 expression also affected tumor initiation and growth *in vivo*, the PCI-13 oral SCC cell line, which was uniformly CD271^+^, was transduced with either a lentivirus that co-expressed the CD271 shRNA and mCherry or a lentivirus expressing GFP. The two sets of transduced cells were mixed at a 1:1 ratio and then injected into immunodeficient Rag^−/−^γc^−/−^ mice (Figure 3). The tumors that formed were subsequently dissociated into single cells and analyzed by FACS, after excluding any infiltrating mouse cells (stained for mouse MHC class I). The tumors were found to be highly enriched for GFP^+^ cells even though the cells that were injected were initially 50% mCherry^+^ and 50% GFP^+^ (Figure 3B). This was the case only when the CD271 shRNA was co-expressed in the mCherry construct. If a an mCherry control lentivirus was used, the resulting tumor remained approximately 50% mCherry^+^ and 50% GFP^+^. Thus, knockdown of CD271 resulted in cells that were significantly less tumorigenic compared to control cells in the same microenvironment, indicating that CD271 is a functional receptor expressed on the tumor-initiating population in SCCHN.

### Targeting CD271 with a monoclonal antibody inhibits *in vivo* tumor formation by SCCHN cells and inhibits phosphorylation of Erk

**Figure 4:**
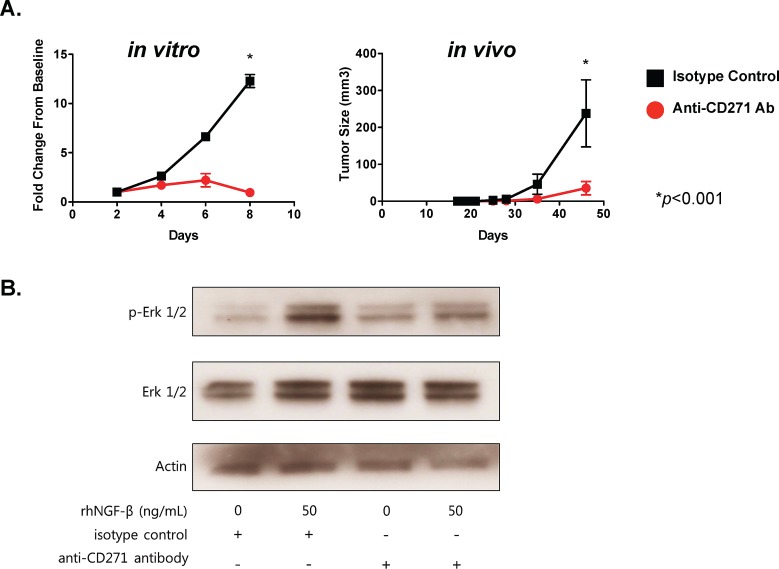
Targeting of CD271 with monoclonal antibody inhibits tumor formation and NGF-induced Erk phosphorylation (A) PCI-13 cells were incubated with either azide-free monoclonal antibody specific for CD271 or isotype control IgG for 30 min, washed, and then assessed for cell proliferation *in vitro* and tumor growth *in vivo*. The graph on the left shows MTT cell viability results over 8 days, expressed as fold-change from the day 0 baseline. The graph on the right shows tumor growth from cells injected subcutaneously into the flanks of Rag^−/−^γc^−/−^ mice. Each cohort consists of 4 mice. Experiments were performed at least two times. (B) PCI-13 cells (grown in serum-free medium for 24 hrs) were incubated *in vitro* with monoclonal antibody to CD271 or isotype control IgG for 30 min and then with or without recombinant human NGF for 1 hr. Cell lysates were subjected to gel electrophoresis, and Western immunoblot analysis was performed with antibody specific for phosphorylated-Erk (p-Erk1/2), total Erk, and actin. All experiments performed two or more times.

The inhibition of *in vivo* tumor formation with CD271 loss-of-function suggested that this molecule might be a viable therapeutic target in SCCHN. To address this hypothesis, we targeted the receptor with a monoclonal antibody specific for NGFR. Incubation of PCI-13 SCCHN cells with the antibody resulted in a significant reduction in cell proliferation *in vitro* compared to cells treated with isotype control IgG (Figure 4A). Furthermore, treatment of the cells with the anti-NGFR antibody resulted in significant reduction in tumor growth *in vivo* compared to isotype control – treated cells.

CD271/NGFR activation has been previously shown to work through downstream activation of both the MAP kinase and PI3 kinase pathways [23]. To determine the functional role of CD271 in SCCHN, we treated PCI-13 cells with recombinant human NGF *in vitro* and assessed activation of the MAP kinase pathway (Figure 4B). NGF treatment resulted in an increase in Erk phosphorylation detected by Western immunoblot. Furthermore, preincubation with the monoclonal antibody to CD271 abrogated this increase in Erk phosphorylation. There was, however, no apparent effect on the PI3 kinase pathway since p-Akt was not affected (data not shown). Our data indicate that CD271/NGFR is functionally active in SCCHN and that targeting CD271 with a monoclonal antibody is a potential therapeutic strategy in this cancer.

## DISCUSSION

In this study, we extend the current understanding and characterization of TICs in SCCHN. We demonstrate that among the CD44^+^ cells in SCCHN, the CD44^+^CD271^+^ cells are the more specific TIC population. In addition, we show that CD271 is a functional cell surface molecule that responds to NGF ligand activation and signals through the MAP kinase pathway. CD271 loss-of-function in these tumor cells results in a significant decrease in their capacity for tumor initiation, and targeting CD271 with a monoclonal antibody results in the inhibition of tumor growth *in vivo*. Thus, our data provide rationale for the targeting of CD271 as a therapeutic strategy.

Our data are consistent with the idea that the TIC population originates from a deregulated normal stem cell population. In normal human oral epithelium, a CD271 expressing subpopulation of cells in the basal layer has been shown to have a stem cell – like phenotype [5]. These CD271^+^ cells have a greater proliferative capacity, form colonies more robustly *in vitro*, and form layers of cells expressing more differentiated markers, resembling stratified epithelium when cultured on amniotic membranes. Furthermore, the incubation of the CD271^+^, but not CD271^−^, cells, isolated from normal human oral epithelium, with NGF resulted in enhanced proliferation. Because CD44 is expressed in the basal half of the oral epithelium, CD271^+^ cells comprise a subset of the CD44^+^ cells in normal epithelium, similar to what we have found in SCCHN. Thus, it is likely that the CD44^+^CD271^+^ cells in SCCHN retain many of the stem cell – like properties of this subpopulation in normal epithelium.

Interestingly, not all of the SCCHN tumors express CD271. This heterogeneity between SCCHN tumors indicates that in some tumors, the TIC population is marked by CD271, but in other tumors, the TIC population may be completely different phenotypically. This may reflect a different cell of origin for the TIC population in CD271^+^ tumors compared to CD271^−^ tumors. It is possible that inciting mutation(s) can occur at different stages of epithelial differentiation and that, consequently, the markers identifying the resulting TIC subpopulation in SCCHN may be different.

Interest in specific strategies to target the TIC population has been gaining traction in recent years with increasing evidence that these cells are more resistant to conventional chemotherapy and radiation therapy in a number of cancer types [24]. In SCCHN, there is evidence that the CD44^+^ population is able to handle oxidative stress better than the CD44^−^ population and that the CD44^+^ cells are enriched after SCCHN xenografts are treated *in vivo* with radiation [2]. Our data would suggest that in CD271^+^ SCCHN tumors, the CD44^+^CD271^+^ cells would comprise the population most resistant to radiation-induced oxidative stress. Supporting this hypothesis is evidence that neurotrophins can increase tolerance of PC12 cells to reactive oxygen species in a CD271-dependent manner [25]. Indeed, in esophageal squamous cell carcinoma, the CD271^+^ cells have been demonstrated to be more resistant to cisplatin, which like radiation, exerts its cytotoxic activity in part through oxidative stress [12, 26]. Since both cisplatin and radiation are mainstay treatment modalities in SCCHN, future work will be focused on assessing the ability of CD271 inhibition to enhance chemoradiation sensitivity of these CD44^+^CD271^+^ TICs.

Multiple studies have observed a correlation between the percentages of TICs, or cancer stem cells, within primary tumors and clinical outcome. In SCCHN, the percent of CD44^+^ cells has been correlated with regional metastatic recurrence [4]. Our data, together with what has been observed in esophageal carcinoma [16, 17], hypopharyngeal carcinoma [14], and oral carcinoma [15, 18], would suggest that the percent of CD44^+^CD271^+^ cells might also provide prognostic information. One of the significant adverse features seen with some SCCHN tumors is perineural invasion (PNI), which has been associated with local recurrence and metastases [27]. Since Schwann cells around neurons and epithelium rich in neurons express NGF [28, 29], it is possible that expression of CD271 may predispose tumor cells to PNI. Indeed, in malignant melanoma, expression of CD271 has been associated with PNI [30], and in oral cancer and pancreatic cancer [31-33], the expression of NGF has also been associated with PNI. In our set of SCCHN samples examined on the TMA, we did not observe an association between CD271 expression and PNI as recorded from pathology reports (data not shown). However, our set consisted of a heterogeneous group of head and neck tumor sites, and further examination of this is needed.

Our data that CD271 signals through the MAP kinase pathway in SCCHN is the first description of an actively signaling receptor that marks TICs in this malignancy. Although CD271 has also been described to signal through the PI3 kinase pathway [34], we did not observe any alterations in Akt phosphorylation with activation or inhibition of CD271 (data not shown). The feasibility of targeting CD271 to affect the viability of CD44^+^CD271^+^ cells in SCCHN and their capacity for tumor initiation is demonstrated by our study. In addition to targeting these cells with monoclonal antibody therapy in vivo, it will be interesting to see if MEK inhibition in these cells can also specifically reduce their viability and/or sensitize them to cisplatin or radiation.

## METHODS

### Formalin Fixed Paraffin-Embedded Tissue Microarray

The tissue microarray (TMA) containing 283 specimens from patient primary tumors was constructed as previously described [35, 36]. These specimens represented a heterogeneous collection of oropharyngeal (tonsil and base of tongue) and non-oropharyngeal (including sites from the oral cavity, hypopharynx, and larynx), retrospectively identified in pathology hospital files. All cases got two punches from areas of tumor that were designated by the study pathologist (JSL) with a dotting pen on the original H&E slides. According to the amount of available biopsied or resected tumor tissue, duplicate two millimeter (or if inadequate tumor tissue present, 0.6 millimeter) punches were taken from each case. Since most of the cases (75%) were treated with primary surgery, the majority of cases on the array had the larger (two millimeter) punches. For the oropharyngeal SCC patients, DNA *in situ* hybridization for high risk human papillomavirus and p16 immunohistochemistry had been previously performed on whole tissue sections, as previously described [37]. CD271 expression was assessed by the study pathologist (JSL) visually and considered simply as a binary result: positive = any tumor cell staining and negative = no tumor cell staining.

### Mice and Cell Lines

Female Rag2^−/−^γc^−/−^ mice (6-12 weeks old) in a Balb/c background were a kind gift from Dr. Irving L. Weissman (Stanford) and were bred in our animal facility under specific pathogen-free conditions. All animal procedures were conducted under institutional guidelines that comply with national laws and policies. The human HNSCC cell line UM-SCC-6 was obtained from Dr. Thomas Carey at the University of Michigan. The oral SCC-4 cell line was obtained from ATCC. The oral oUPCI:SCC103 and PCI-13 cell lines were kind gifts from Dr. Suzanne Gollin Theresa Whiteside and Dr. Jennifer Grandis at the University of Pittsburgh. Cells were maintained in complete DMEM:F12 medium (DMEM:F12 1:1 with Glutamax [Gibco, Invitrogen, CA] containing: 10% heat-inactivated FBS [Cellgro, MA], 100 IU/ml penicillin and 100 μg/ml streptomycin [Gibco, Invitrogen, CA]). The HEK293T cell line was obtained from ATCC and maintained in complete DMEM medium. The MOC2 and MOC10 murine oral SCC cell lines were developed by Dr. Ravindra Uppaluri at Washington University [38].

### Antibodies

Monoclonal antibodies to CD31 (WM-59), CD45 (30-F11), CD44 (G44-26), CD271 (C40-1457) for flow cytometry and sorting were obtained from BD Biosciences. Antibody to mouse CD271 (mu p75) was obtained from Advanced Targeting Systems. Antibody to fibroblasts (TE-7) was obtained from Millipore. Anti-mouse H-2K^d^ antibody (SF1-1.1) was obtained from BD Biosciences. Antibody to CD271 (NGFR5) for immunohistochemistry and blocking experiments was obtained from Abcam and Thermo Scientific. Antibodies to phospho-p44/42 MAPK (Erk1/2) (Thr202/Tyr204) (E10) and total Erk were obtained from Cell Signaling Technologies. Antibody to actin was obtained from Thermo Scientific.

### Tumor Digestion and *In Vivo* Xenograft Assays

Patient tumor samples were obtained fresh from the operating room after a consenting process that was approved by the Institutional Review Board. Tumor cell dissociation was performed, as follows. Briefly, tumors were minced and then digested with collagenase-hyaluronidase (Stem Cell Technologies) for 16 hrs at 37 deg C. Following this, the cells were treated for 3 min with trypsin-EDTA, washed, treated with dispase and DNase I (Stem Cell Technologies) for 1 min, washed, filtered, and resuspended in PBS or FACS buffer.

For *in vivo* xenograft assays, female Rag2^−/−^γc^−/−^ mice were injected subcutaneously with the indicated number of viable cells, after cell dissociation, washing, and suspension in PBS. Tumor growth was monitored twice a week and tumor diameters were measured using an electronic calliper to determine the tumor size by multiplying perpendicular diameters.

### Quantitative RT-PCR

The relative abundance of p75^NTR^ mRNA was analysed by quantitative real-time reverse transcription-Polymerase Chain Reaction (RT-PCR) of SCC-4, UM-SCC-6, UPCI:SCC-103 and PCI-13 cells. Briefly, cell cultures were rinsed with ice-cold PBS and then the cells were lysed and scraped off using Trizol (Invitrogen). Afterwards, chloroform (Sigma) was added and the mixture was centrifuged. RNA was recovered from the aqueous phase by precipitation with 2-propanol. The extracted RNA was treated with Turbo DNase to remove genomic DNA prior to reverse transcription with Superscript III Reverse Transcriptase (Invitrogen). Finally, real-time RT-PCR was performed using a specific Taqman Assay for the human p75^NTR^ sequence. Human HPRT1 was amplified as control. The amount of p75^NTR^ mRNA was expressed as arbitrary units defined as the n-fold difference relative to the control gene *HPRT1* (2ΔCt ×100, where ΔCt represents the difference in threshold cycle between the control and target genes).

### Lentiviral Plasmids

The lenti-vectors pLKO.1 puro and pLKO.1 mCherry were kind gifts from Dr. Alejandro Sweet-Cordero (Stanford). The following shRNAs against p75^NTR^ were AgeI/EcoRI inserted into the pLKO.1 vectors: shp75NTR1(5′CCGGGCAC TGTAGT AAATGGCA ATTCTCG AGAATT GCCATTTACTACAGT GCTTTTTG3′),shp75NTR2(5′CCGGCCTCCAGAACA AGACCTCATACTCGAGTATGAGGT CTTGTTCTGGAGGTTTTTG3′),shp75NTR3(5′CCGGCCAGCC TAAGATGAAGAGGATCTCGAGA TCCTCTTCATCTTAGGCTGGTTTTTG3′),shp75NTR4(5′CCGGCCTTCA CTTCTGACCACACTTCTCGAGA AGTGTGGTCAGAAGTGAAGGTTTTTG3′),shp75NTR5(5′CCGGGCGGCA AGAAGGAATTGACTTCTCGAGAAGTC AATTCCTTCTTGCCGCTTTTTG3′).

The lentiviral constructs pHIV-Zsgreen and pHIV-mCherry were kind gifts from Dr. Michael Clarke (Stanford). A cDNA from the ATG codon to the stop codon of human p75^NTR^ was PCR cloned (forward primer: 5′CATTAGCGGCCGC ACCATGGGGGCAGGTGCC-3′; reverse primer:5′CTGGTCTAGATCACACC GGGGATGTGGCAGTG-3′) and NotI/XbaI inserted into the pHIV-Zsgreen vector.

### Lentiviral production

For the production of the lentiviral particles, the cell line HEK293T was transfected, using CaCl_2_, with the packaging plasmid pCMV-dR8.2, the envelope plasmid pCMV-VSVG and the lentiviral construct containing the shRNA or the transgene. Cell culture medium was changed 16 hours after the transfection and virus supernatants were collected 24 and 48 hours after the media change. Immediately after supernatant collection, the viral particles were concentrated by ultracentrifugation. The lentiviral pellets were then resuspended in ice-cold PBS and the virus was titrated by FACS using HEK293T cells. To calculate the biological titer of the virus, the following formula was used: TU/μl = (P × N/Vx100) × 1/DF, where TU stands for Transducing Units, P = % Zsgreen^+^ or mCherry^+^ cells, N = number of cells at time of transduction = 10^5, V= volume of dilution added to each well = 20 μl, and DF = dilution factor = 1 (undiluted), 10^−1 (diluted 1/10), 10^−2 (diluted 1/100), and so on.

### Lentiviral transduction

For the lentiviral transduction of the cell lines, cells were harvested, washed, resuspended in fresh medium and plated at the appropriate concentration (1×10^6^ cells per 10 cm plates). Then, the lentiviral particles were added to the cell cultures at a multiplicity of infection (MOI) of 1 transducing Unit per cell. Polybrene (8ug/ml) was also added to enhance the lentiviral transduction efficiency. 48 hours after viral infection, medium was changed. In the case of the cells transduced with the pLKO.1 puro vectors, the cell cultures were treated with the selection agent puromycin for 3 days after media change.

### XTT and MTT Assays

The XTT and MTT assays were carried out following the manufacturer's instructions (Roche Applied Science, Germany). Briefly, FACS-sorted cells were seeded into 96-well plates at a concentration of 500 to 1500 cells per well in 100ul of culture medium. The XTT labelling mixture was added to the wells at different time points and incubated for 20 hours at 37 C before reading the plate in an ELISA reader at a wavelength of 450-500 nm. For the MTT assays, MTT labelling reagent was added to the wells first. Then the plates were incubated for 4 hours at 37 C and finally the solubilisation buffer was added to the wells and incubated at 37C for another 16 hours before evaluating the plate in an ELISA reader at a wavelength of 550-600nm.

### CD271 Rescue Experiment

To validate the selectivity of the shRNAs 3a and 5a for p75^NTR^ gene, a rescue experiment was performed employing an expression construct containing a p75^NTR^ cDNA that was resistant to the shRNAs. Briefly, PCI-13 cells were transduced first with the lenti-vector pHIV-Zsgreen containing the cDNA of human p75^NTR^ that lacked the 3′UTR region. 5 days after transduction, Zsgreen^+^ cells were sorted out by FACS. Afterwards, these cells were co-transduced with the shRNAs 3a or 5a that were designed to the 3′UTR region of p75^NTR^ mRNA. 5 days later, cells were FACS-sorted again based on the expression of Zsgeen and mCherry. The double-sorted cells were then plated into 96-well plates (500-1500 cells per well) to carry out a XTT or MTT assay.

### *In vivo* Tumor Growth Competition Assay

For the competition assay, the cell line PCI-13 was transduced with the lentiviral construct pHIV-Zsgreen (green) or with the *lenti*-vector pLKO.1 mCherry (red) containing or not the specific shRNAs against p75^NTR^ 3a and 5a. Two days later, the culture medium was changed and five days after the viral transduction, the cells were harvested and washed twice with ice-cold PBS. The single-cell suspensions were then FACS-sorted based on the expression of the reporter molecules Zsgreen (green) and mCherry (red). The double-sorted red and green cells were mixed in similar amounts and injected subcutaneously into immunocompromised Rag2^−/−^γc^−/−^ mice. The tumors that arose in those mice were harvested when they reached 1.5 cm in diameter and were dissociated using collagenase-hyaluronidase from StemCell Technologies following the manufacturer's instructions. Finally, the dissociated-tumors were analysed by flow cytometry.

### Flow Cytometry Analysis

Cells were harvested from culture flasks or isolated from mouse xenografts after tumor dissociation, as described above. Single-cell suspensions were then pre-treated with IgG from mouse serum to block non-specific staining and incubated with the appropriate antibodies. DAPI was used to allow exclusion of non-viable cells; in the case of mouse xenografts, anti-mouse H-2K^d^ FITC antibody (SF1-1.1) was added to exclude mouse cells from the analysis. Then, CD44 and CD271 expression was assessed after exclusion of lineage^+^ cells (CD45^+^ cells, CD31^+^ cells, and TE-7^+^ fibroblasts).

To determine the percentage of cells within the PCI-13 cell line that were actively undergoing apoptosis after p75^NTR^ knock down, the FITC Annexin V Kit from BD Pharmingen was used following the manufacturer's instructions. Briefly, cells were washed twice in ice-cold PBS and resuspended in 1x binding buffer at a concentration of 1×10^6^ cells/ml. The cells were then incubated in the presence of Annexin V and PI for 15 minutes at room temperature in the dark. After the 15-minute incubation, 400ul of 1x binding buffer was added to each tube and the samples were analysed within 1 hour.

### Cell Cycle Analysis

For the cell cycle analysis, the oral squamous cancer cell line PCI-13 was transduced with lentiviral vectors carrying the specific shRNAs against p75^NTR^ shRNA 3a or shRNA 5a or with the empty lenti-vector pLKO.1. puro. Two days after the viral transduction, the selective agent puromycin was added to get rid of the untransduced cells. After 3 days in the presence of the selection drug, the medium was changed and BrdU was added to the cell cultures for 4 hours. After the 4-hour incubation, the cells were harvested and washed with PBS twice to remove the non-incorporated BrdU. Then the BrdU labelled cells were fixed with 70% ethanol and kept at -20 degrees for at least 2 hours. The day of the experiment, the cells were thawed and rehydrated in PBS for 15 minutes. DNA was denatured by adding drop-wise a solution containing 2M HCl and 0.5% of Triton-X100. After a 20-minute incubation, the acid solution was neutralized using 0.1M sodium borate at pH 8.5. Finally, the samples were incubated in the presence of anti-BrdU FITC antibody (BD Pharmingen) for 30 minutes, washed and added a solution containing PI and RNase A just before they were analysed by FACS.

### Targeting CD271 *in vitro* and *in vivo*

PCI-13 cells were incubated with either azide-free monoclonal antibody specific for CD271 (clone NGFR5, Abcam, 5 ug/ml) or azide-free isotype control IgG (BD Biosciences, 5 ug/ml) for 30 min, washed, and then assessed for cell proliferation *in vitro* and tumor growth *in vivo*. The *in vitro* MTT cell viability results were assessed over 8 days and expressed as fold-change from the day 0 baseline. For the *in vivo* tumor growth assessments, cells were injected subcutaneously (100,000 cells per mouse) into the flanks of Rag^−/−^γc^−/−^ mice. Each cohort consisted of 4 mice. The experiments were done a minimum of two times.

### Western Immunoblot

PCI-13 cells were incubated with serum-free DMEM medium for 24 hrs. The cells were incubated with either monoclonal antibody to CD271 (NGFR5, Thermo Scientific) for 30 min and then, incubated with recombinant human NGF (Shenandoah Biotechnology, Inc.) for 1 hr. Whole cell lysates were collected, and proteins were separated by gel electrophoresis. Western analysis was performed with phospho-Erk, total Erk, and actin antibodies, described above.

### Statistical Analysis

Prism software (Graph Pad Software, Inc.) was used to analyse tumor growth, cell viability, cell cycle, apoptosis and for the competition analysis *in vivo* and to determine statistical significance of the differences between groups by applying unpaired Student's t-tests or U-Mann Whitney tests. P values of <0.05 were considered significant.

## SUPPLEMENTARY MATERIAL FIGURES


