# Supplementary material for: CD271 is a functional and targetable marker of tumor-initiating cells in head and neck squamous cell carcinoma
Source: Oncotarget. 2014 Jul 26;5(16):6854–66. doi: 10.18632/oncotarget.2269 (PMC4196168; doi:10.18632/oncotarget.2269)
Supplement: Supplementary file 1 [file oncotarget-05-6854-s001.pdf]

CD271 is a functional and targetable marker of tumor-initiating cells in head and neck squamous cell carcinoma

Supplementary Material

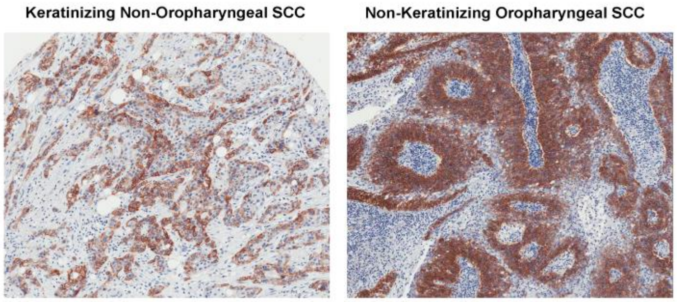

Supplemental Figure 1: Examples of CD271 staining in formalin-fixed paraffin-embedded tissue from keratinizing non-oropharyngeal SCC and non-keratinizing oropharyngeal SCC. Human primary SCCHN samples were stained with a monoclonal antibody against CD271 (clone NGFR5) and assessed by immunohistochemistry.

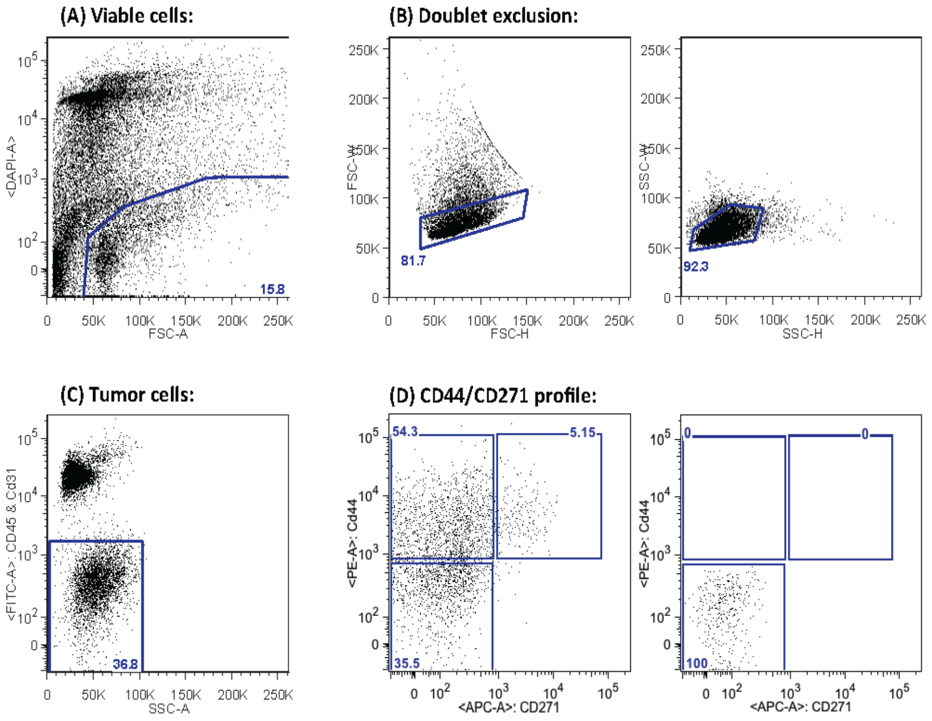

Supplemental Figure 2: Gating strategy for sorting CD44/CD271 populations from patient samples for *in vivo* limiting dilution tumor growth assays. Cells are gated sequentially in the order shown. DAPI-negative viable cells are selected (A), then forward scatter and side scatter height *vs*. width plots are used to exclude doublets (B). Lineage-positive cells are excluded (C), after which the CD44 *vs*. CD271 profile is obtained (D; left). CD44/CD271 gates are set based on control stained cells (D; right).

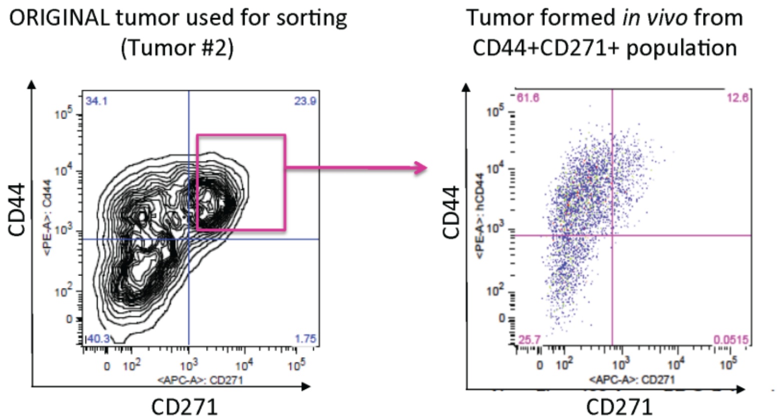

Supplemental Figure 3: $CD44^+CD271^+$ cells recapitulate the heterogeneity of the parent tumor after tumor formation and passage in mice. The FACS plot on the left shows the CD44 and CD271 staining of the parent tumor after gating out DAPI-positive and lineage-positive cells. The FACS plot on the right shows the profile of the tumor arising after implantation of purified $CD44^+CD271^+$ cells in mice and *in vivo* passage.

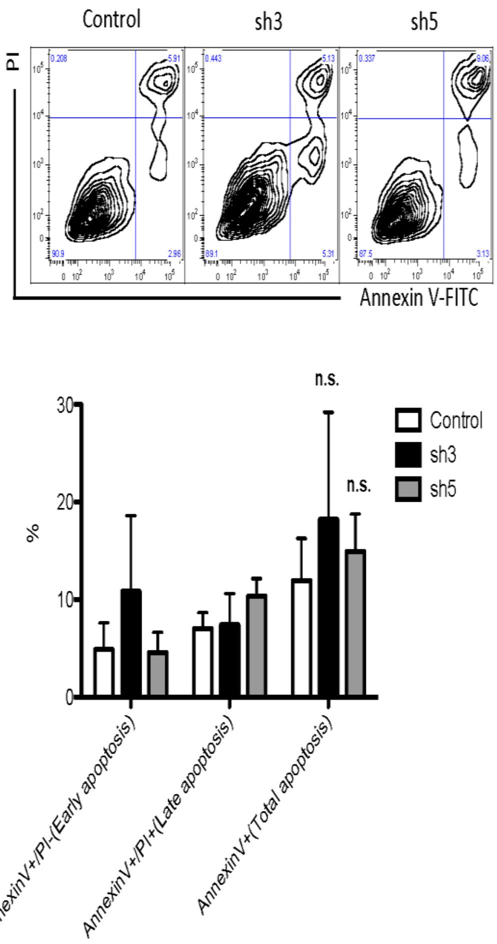

Supplemental Figure 4: CD271 loss-of-function does not increase the rate of apoptosis in oral SCC. Knockdown of CD271 in the human oral SCC cell line PCI-13 was accomplished by lentiviral transduction of shRNA specific for CD271. Initiation of apoptosis and cell death was assessed by annexin V and propidium iodide (PI) co-staining and FACS. Two different shRNAs were assessed: “sh3” = CD271 shRNA3. “sh5” = CD271 shRNA5. Control = scrambled RNA.
